# Commentary: Engaging healthcare staff and stakeholders in healthcare simulation modeling to better translate research into health impact: A systematic review

**DOI:** 10.3389/frhs.2022.982184

**Published:** 2022-08-19

**Authors:** Bo Kim

**Affiliations:** ^1^Center for Healthcare Organization and Implementation Research, VA Boston Healthcare System, Boston, MA, United States; ^2^Department of Psychiatry, Harvard Medical School, Boston, MA, United States

**Keywords:** commentary, translation, simulation model, participatory research, healthcare improvement, community engagement

## Introduction

As simulation modeling is increasingly embraced for implementation research, Zabell et al. offer a very timely and thought-provoking systematic review. The review comprehensively synthesizes the field's knowledge regarding the processes and impacts of engaging in simulation modeling those who are affecting or affected by an innovation's implementation [henceforth, implementation “associates”; please note this use of an alternative term for “stakeholders” for this commentary, per (1)]. Notable strengths of the review article include its identification of potential mechanisms through which specific processes used for engagement affect successful model development and usage, as well as its guidance on existing frameworks that can be leveraged for improved reporting and evaluation of engagement processes. The article urges future research around validated measures-driven evaluation of engagement for simulation modeling, and it also appropriately recognizes the likely changes to engagement process considerations since the COVID-19 pandemic (the timeframe of which is not included in the review). By highlighting and expanding on these visions for future work that the article discusses, this commentary aims to set the stage upon which the field can contextualize and debate the necessity and importance of the envisioned future work for advancements in associate engagement for simulation modeling.

## Evaluation of associate engagement for simulation modeling

For enhanced reporting of associate engagement processes for simulation modeling, the authors discuss the potential combined use of Gray et al.'s four-dimensional reporting framework (4P) for standardized reporting of associate engagement (2) and Baldwin et al.'s Modeling Approach that is Participatory Iterative for Understanding (MAPIU) for associate-engaged simulation modeling (3). For addressing the authors' call for future work on devising systematic approaches to evaluating the processes that are specified (for instance, using 4P and MAPIU), an additional framework to consider may be the list of key evaluation questions (Table 1) that Goodman and Sanders Thompson recommend in their 2017 commentary on the science of associate engagement in research (4). The questions include asking which associates will benefit from what engagement processes, and how the implementation research team would know the quantity and quality of associate engagement. Goodman and Sanders Thompson posit that considering these questions enables research teams to pursue meaningful associate engagement that creates “an amalgam for research synergy allowing the partnership [e.g., between the research team and associates] to obtain outcomes that no one constituent member could have produced on their own (4).” Hence, considering these questions for evaluating associate engagement processes may enable gauging the extent to which the processes contribute to establishing such synergy around simulation modeling. Importantly, Goodman and Sanders Thompson encourage teams to begin considering these questions early in their planning of associate engagement, which precisely aligns with Zabell et al.'s recommendation that evaluation of associate engagement for simulation modeling “should be an *a priori* embedded component of the research design.”

**Table 1:** Key questions for conducting and evaluating associate engagement, adapted from (4).

Who will benefit from the implementation effort?
Who are the appropriate associates to engage?
What is the current nature/extent of associate engagement?
What is the desired nature/extent of associate engagement?
What processes should be developed and used for reaching and sustaining the desired nature/extent of associate engagement?
If multiple associates participate and only a subset benefits, was the engagement meaningful or perfunctory?
How will the quality and quantity of associate engagement be evaluated?
Does associate engagement improve the evidence-based practice's fit with the implementation setting(s)?

## Considerations for associate engagement under COVID-19

The authors acknowledge that approaches to and considerations surrounding associate engagement processes are expected to be different for simulation modeling efforts under COVID-19. The differences are likely to be in both how the processes are conducted (e.g., virtual components replacing face-to-face components) and the nature of the implementation tasks at hand (e.g., crisis management of a public health emergency). Not specific to simulation modeling, a considerable collection of literature is developing around associate engagement for implementation efforts that target underserved communities that the pandemic disproportionately affected. For example, particularly for community engagement, den Broeder et al. propose specific features of engagement that are crucial for health promotion success during COVID-19 (5), and Corbin et al. report on a global multiple-case study that identified engagement activities that can help mitigate the consequences of public health emergencies and other crises (6). Especially as Zabell et al. mention that examining associate engagement for simulation modeling in the COVID-19 context “is the subject of a separate subsequent project,” it will be of great interest to learn from their subsequent work whether how engagement processes account for COVID-19 is different for simulation modeling efforts vs. for implementation-related efforts more generally that involve associate engagement.

## Reviews of central aspects of simulation modeling beyond associate engagement

Findings of the systematic review—namely, that associate engagement processes used for simulation modeling “are heterogeneous and often based on intuition rather than clear methodological frameworks”—suggest the potential benefit of conducting analogous comprehensive reviews of other central aspects of simulation modeling beyond associate engagement. Especially of interest may be aspects of simulation modeling for which this review found limited examples of associate engagement, such as computer model building. The various and dynamic real-world contexts and implementation efforts that use simulation modeling may necessitate heterogeneous approaches to computer model building. However, it may still be worth examining how modelers select the appropriate computational representation of a conceptualized model, handle uncertainties in the model, and visually represent or methodically document the model. For instance, to what extent do modelers align to established principles and best practices for each of these steps in building the model (7), including looking to other fields outside of healthcare for which modeling has historically been more widely used? Variations in approaches regarding these steps, as do variations in associate engagement processes, may affect the validity, utility, and impact of simulation modeling in translating research into health impact.

## Discussion

Zabell et al.'s systematic review comes at a time when there is a growing and urgent call for knowledge translation efforts to better meet the needs of unique contexts into which evidence-based practices are implemented. As implementation research actively incorporates innovative applications of promising methods (such as simulation modeling), to help answer this call, careful assessments of the methods' value added (including the preparatory steps needed to allow for such assessments) must be delineated and shared across the field. For associate engagement, which is deeply rooted in theories and practices of organizational, management, and behavioral sciences, there is an opportunity for implementation science to learn from how those fields have incorporated associate engagement into applying innovative methods in their research. An example of such learning is Elwy et al.'s approach to selecting specific engagement strategies for different types of associates (e.g., supportive and non-supportive) when disseminating research information that is central to successful implementation (8), which draws on Freeman's theory that an organization's success depends on its ability to create value for all of its associates (9).

Through the aforementioned strengths of their systematic review and the directions for meaningful future work that the review encourages, the authors provide a useful roadmap that similar efforts can follow to synthesize existing knowledge regarding, and prepare for rigorous assessments of, innovative methods for implementation research. Heterogeneity of evidence-based practices and their implementation contexts likely require applications of innovative methods to be heterogeneous as well, pointing to the need for standardized yet flexible structures and guidelines that make their applications and assessments both comparable across implementation efforts and adaptable to specific cases of implementation. Similar to the field's focus on determining not only what works for implementation but also for whom and how, informative assessments of innovative methods should elucidate the circumstances under which the methods are more or less applicable.

## Author contributions

BK conceptualized and wrote the commentary, inspired by Zabell et al.'s article and valuable implementation research collaborations.

## Conflict of interest

The author declares that the research was conducted in the absence of any commercial or financial relationships that could be construed as a potential conflict of interest.

## Publisher's note

All claims expressed in this article are solely those of the authors and do not necessarily represent those of their affiliated organizations, or those of the publisher, the editors and the reviewers. Any product that may be evaluated in this article, or claim that may be made by its manufacturer, is not guaranteed or endorsed by the publisher.
